# A young woman with atypical McCune–Albright syndrome and the difficult road to recovery: a case report

**DOI:** 10.3389/fsurg.2024.1326977

**Published:** 2024-02-02

**Authors:** Hongbin Wang, Hao Wang, Heng Liu, Xin Yang, Zhichao Meng, Yongping Cao

**Affiliations:** Department of Orthopedics, Peking University First Hospital, Beijing, China

**Keywords:** McCune-Albright syndrome, bisphosphonate, fracture, arthroplasty, complication

## Abstract

**Background:**

Fiber dysplasia is a complex condition that presents with various clinical manifestations, such as deformity, dysfunction, pathological fractures, and endocrine disorders. McCune–Albright syndrome (MAS) is a rare subtype of fiber dysplasia. This article reports a case of atypical McCune–Albright syndrome in a patient with a femoral neck fracture.

**Case presentation:**

A patient with atypical McCune–Albright syndrome sustained a right femoral neck fracture and underwent multiple treatments, including total hip replacement, intravenous infusion of zoledronic acid, oral calcium supplementation, right supracondylar osteotomy, orthopedic surgery, plate and screw internal fixation for a left femoral shaft fracture, and removal of the right femoral plate. The patient also developed a submaxillary infection complicated by mandibular osteonecrosis.

**Conclusion:**

Patients with MAS may experience rare complications as a result of their unique condition, regardless of whether they receive drug or surgical treatment. Therefore, personalized drug regimens and feasible surgical options are necessary.

## Case report

In December 2009, a 24-year-old female patient was admitted to the hospital with complaints of “right hip pain and limited mobility for over a year following a fall.” The patient experienced right hip pain after a fall that occurred more than a year ago. The pain experienced limited movement. After resting, the pain was slightly relieved, and she was able to walk.

**Figure 1:**
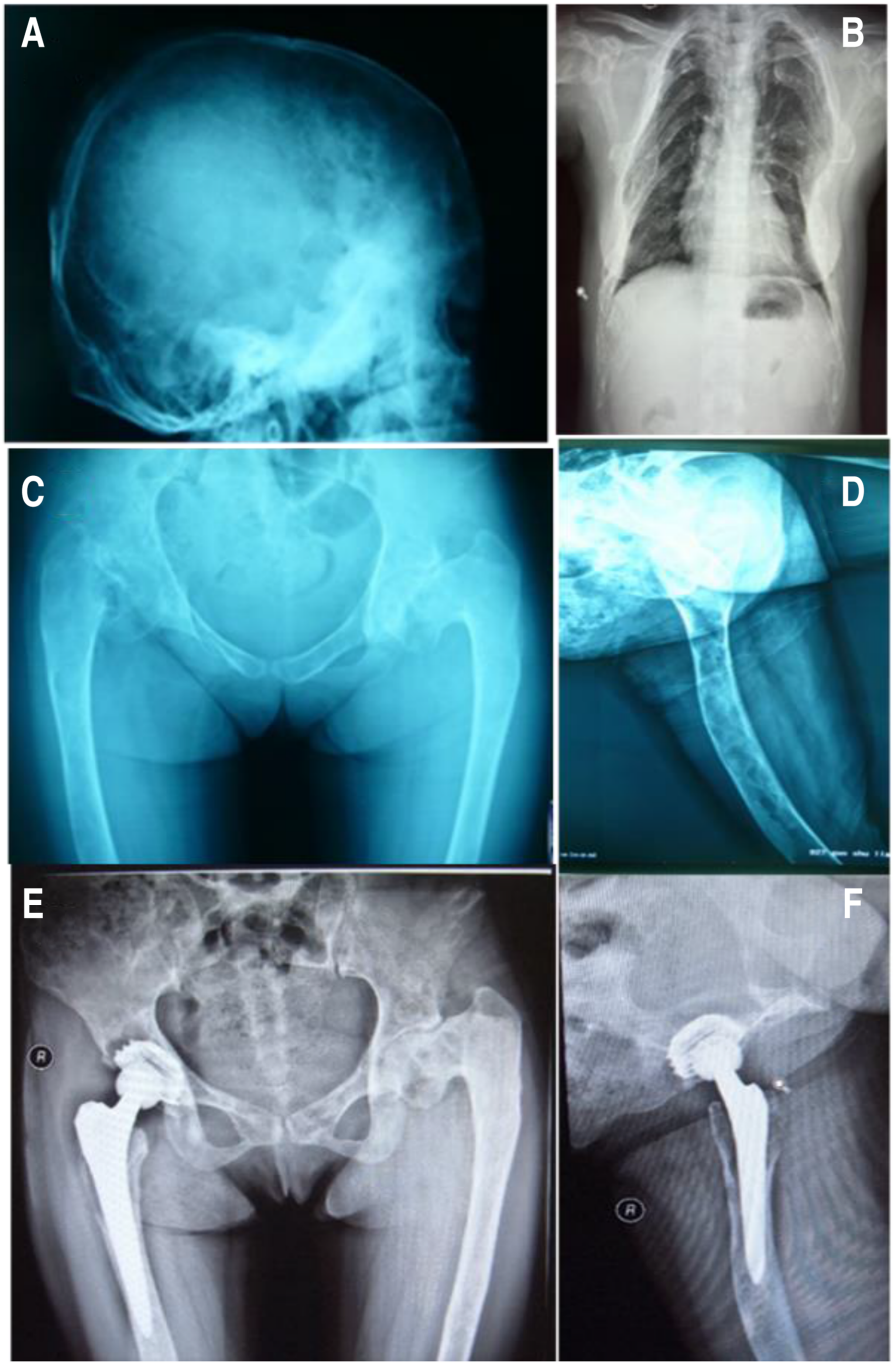
X-rays of the patient on initial admission. ABCD: Preoperative radiograph, EF: 22 months after right hip replacement.

According to the provided information, the patient had a 10-year history of multiple untreated skeletal abnormalities, which included double hip varus deformity, flexion contracture, and adduction deformity of the right hip and bilateral knee valgus deformity. Multiple cystic areas of bone densification and ground glass density in both the lower limb long bones and skull, thinning of the bone cortex and deformation of the bilateral femoral shaft and cervical trunk angle were discovered via x-ray on admission (Figure 1). Additionally, the patient had multiple skin pigmentations at birth (Supplementary Material) and was diagnosed with a facial bone deformity at the age of 14. However, she had normal menstruation and secondary sexual characteristics and delivered a child naturally at the age of 24.

The patient also had abnormal blood biochemistry findings, with markedly increased levels of serum alkaline phosphatase (670 IU/L, normal value: 40–160 IU/L) and osteocalcin (219.6 ng/ml, normal value: 11–43 ng/ml). However, her serum calcium concentration was within the reference range, and other endocrine indices were not abnormal (Supplementary Material).

The diagnosis considered based on these findings is McCune–Albright syndrome (MAS), a rare genetic disorder characterized by the presence of fibrous dysplasia, endocrine abnormalities, and cafe-au-lait spots on the skin. In this case, the patient had skeletal abnormalities consistent with fibrous dysplasia, as well as elevated levels of alkaline phosphatase and osteocalcin.

**Figure 2:**
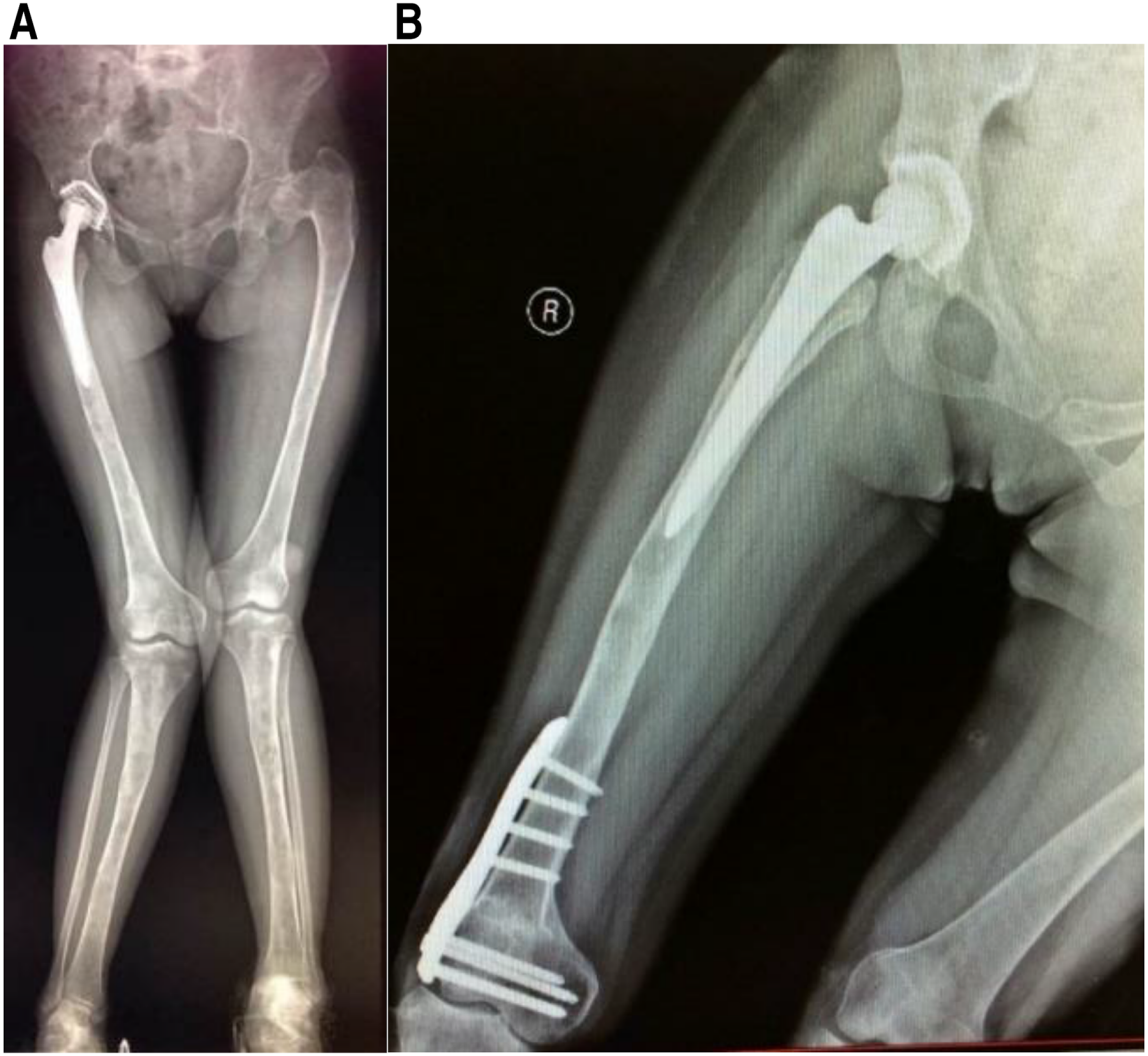
(**A**) Preoperative full-length lower limb radiograph. (**B**) X-ray after right knee valgus correction.

**Figure 3:**
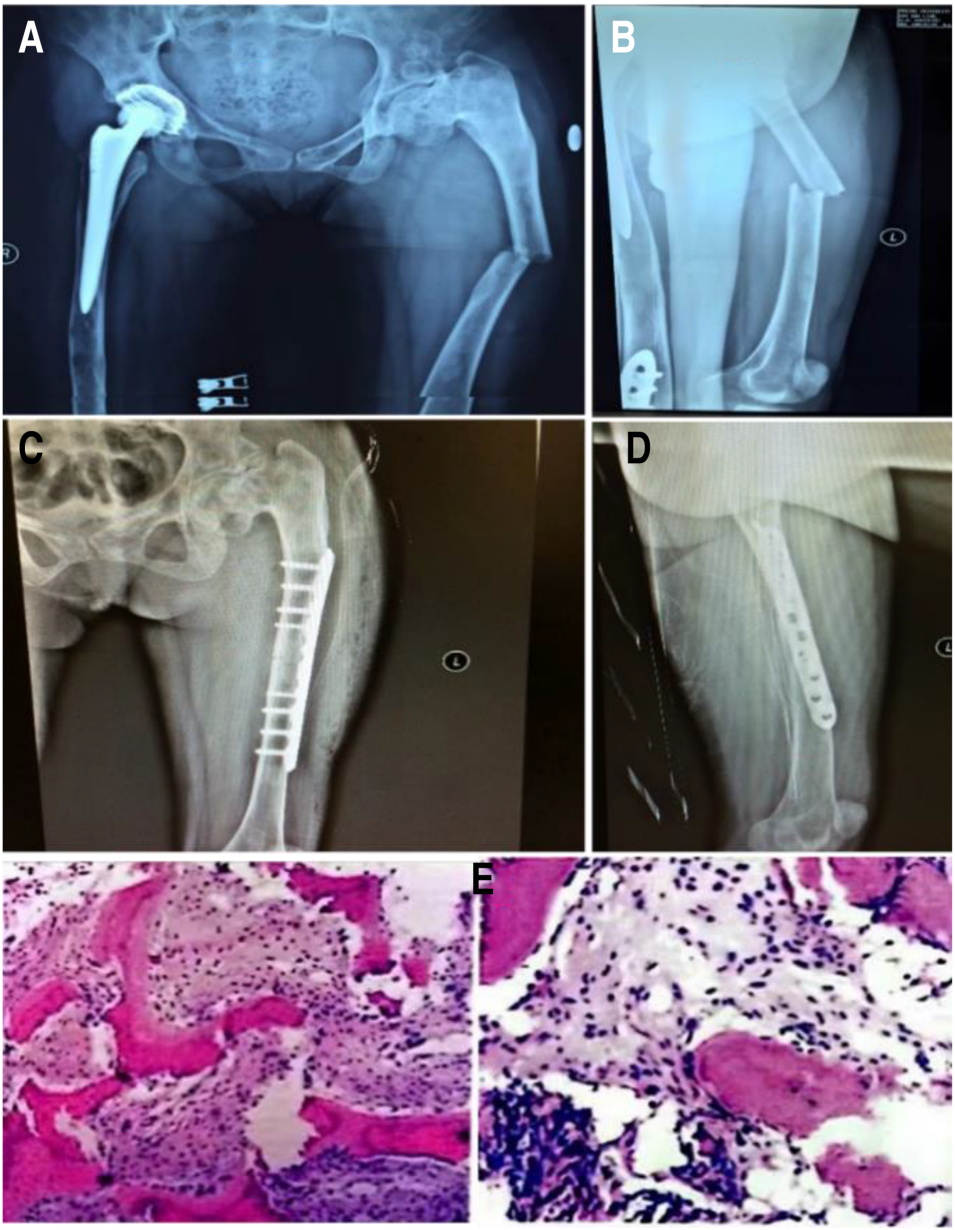
Left femoral shaft fracture under slight external force. (**A,B**) Preoperative x-rays, (**C,D**) postoperative x-rays, (**E**) postoperative pathology.

**Figure 4:**
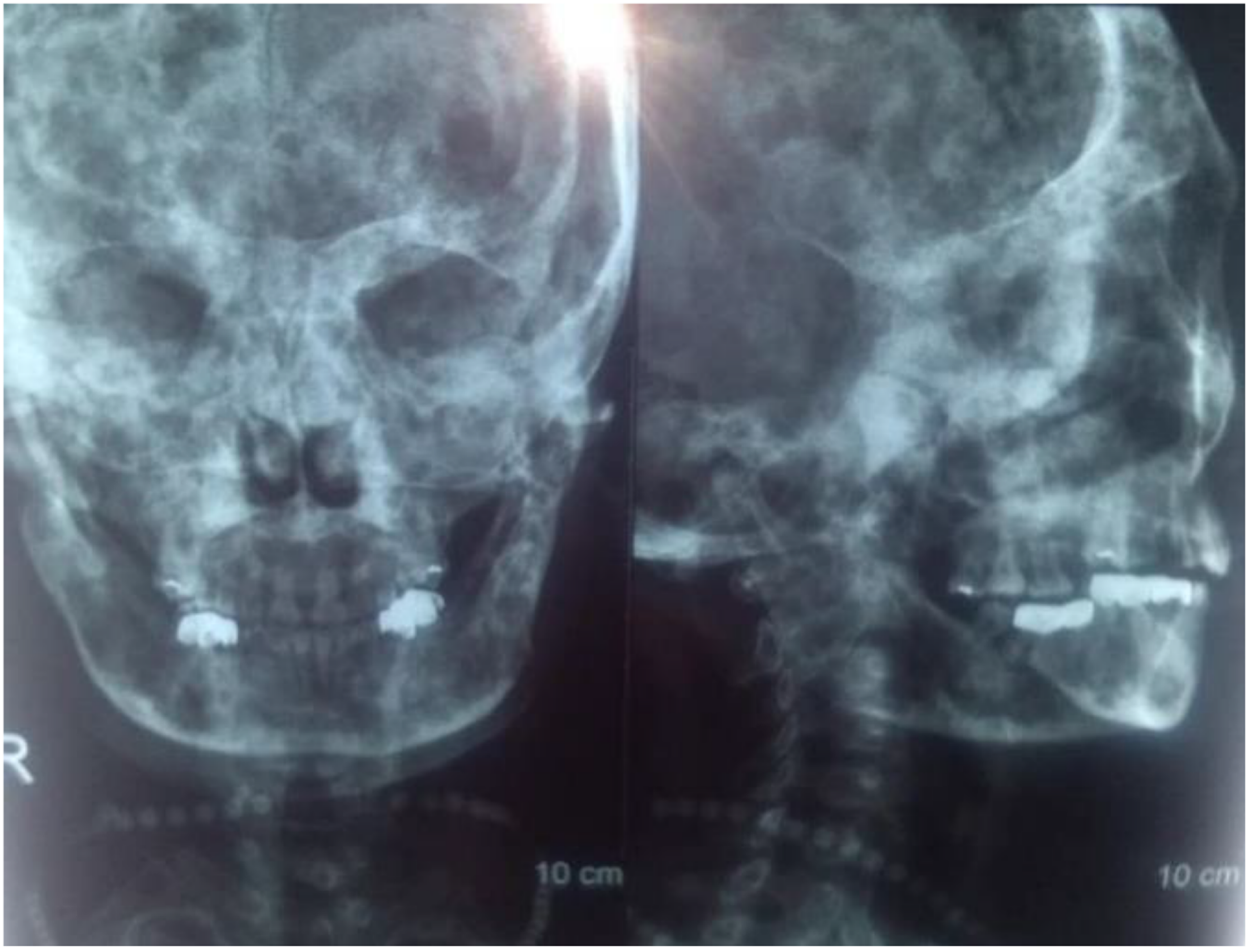
X-rays of osteonecrosis of the mandible.

The patient received 5 mg of zoledronic acid through intravenous infusion four times between 2009 and 2013, along with oral calcium supplementation. Total hip replacement (THA) was performed on the right side in July 2010. Postoperative hip radiographs were examined (Figure 1). The alkaline phosphatase and osteocalcin levels were 538 IU/L and 192.5 ng/ml, respectively. In December 2013, the patient underwent orthopedic surgery and a right supracondylar osteotomy (Figure 2). Upon admission, the alkaline phosphatase and osteocalcin levels were 403 IU/L and 246.30 ng/ml, respectively. In May 2015, the patient sustained a left femoral shaft fracture due to a fall and underwent internal fixation with plates and screws (Figure 3). Based on postoperative histopathology, fibrous dysplasia was ultimately diagnosed. In December 2015, the right femoral plate was removed to prevent stress concentration and subsequent fracture in the femoral shaft. Although the patient had no significant complications during the follow-up period, she was treated for submaxillary infection and mandibular osteonecrosis at another hospital in 2017 and 2018 (Figure 4). The difficult road to recovery of this patient was shown in the Table 1.

**Table 1:** The course of the patient's treatment.

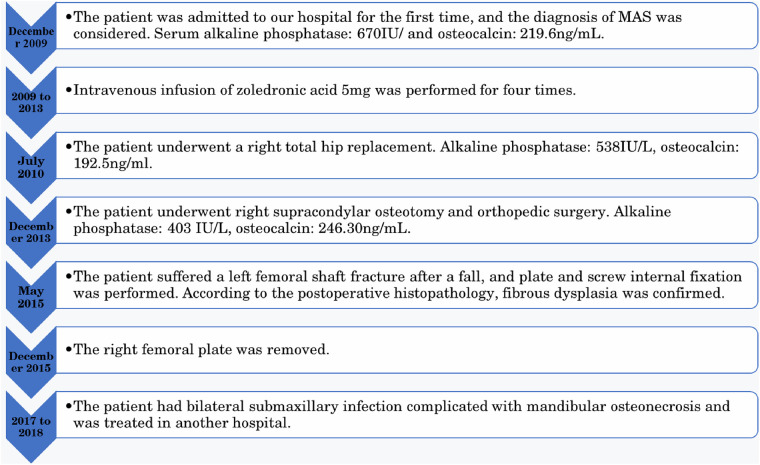

## Discussion

McCune–Albright syndrome (MAS) is an uncommon endocrine and metabolic disorder that was initially described by McCune and Albright in 1937. This condition occurs sporadically and can manifest at any age, ranging from 4 months to 70 years, with a higher prevalence among women. Typical MAS presents with singular or multiple fibrodysplasia, café-au-lait spots on the skin periphery, and associated endocrine irregularities. Lumbroso et al. (1) reported that MAS exhibit a characteristic triad (24%), tandem signs (33%), and a single manifestation (40%). When symptoms deviate from the typical presentation, misdiagnosis or delayed diagnosis can occur. The phenotype of MAS is highly varied, with x-ray features including ground glass changes, luffa fascia changes, cystic alterations, and insect erosive changes. Some atypical x-ray features can be challenging to differentiate from conditions such as bone cysts, Paget's disease of the bone, and bone tumors (2). Additionally, the diagnostic utility of these methods is limited by the lack of helpful indices and poor specificity. The most distinguishing finding in our patient's case was the significant increase in alkaline phosphatase and osteocalcin levels. However, patients with Paget's disease of the bone can also exhibit substantial abnormalities in these markers. Consequently, histopathological examination is often the ultimate means of disease diagnosis. Nonetheless, distinguishing between fibrous dysplasia (FD) and osteofibrous dysplasia is difficult, as these conditions share similar histological features. Advanced imaging techniques such as CT, MRI, and PET-CT may prove invaluable in challenging identification scenarios.

At present, there is no specific treatment for MAS. The primary focus is on early diagnosis and addressing any concurrent endocrine disorders. Nonsurgical treatment with bisphosphonates is commonly employed for symptomatic FD in patients diagnosed with MAS. Research indicates that an abundance of osteoclasts and bone resorption are typical features of FD lesions (3). Bisphosphonates can hinder osteoclast activity, reduce bone turnover, stimulate osteogenesis, and retard or even reverse the early stages of osteolysis, thus effectively relaying disease progression. However, the body of literature on the therapeutic effects of bisphosphonates yields inconsistent findings (4–7). Moreover, bisphosphonate therapy can lead to a spectrum of adverse reactions, including myalgia, flu-like symptoms, gastrointestinal side effects, an elevated risk of atrial fibrillation, and rare complications such as atypical fractures and osteonecrosis (8).

In this case, the patient received bisphosphonate therapy only four times over five years, and the treatment was discontinued after favorable results were achieved. In the second year following cessation, the patient sustained a minor femoral shaft fracture due to external force. We hypothesized that fibrous dysplasia (FD) was the underlying cause of this pathological fracture. It is important to differentiate this condition from atypical femoral fractures (AFFs) induced by bisphosphonates. AFFs typically manifest approximately 5–6 years after commencing bisphosphonate treatment, although some cases have been reported within 1–2 years of treatment initiation (9). AFFs often present with lateral cortex thickening at the fracture site, accompanied by a periosteal reaction. Other characteristic features include prodromal pain, bilateral fractures, and transverse fractures, none of which align with the patient's condition. The specific etiology of AFFs has not been determined, but one potential mechanism is the inhibition of bone turnover, which can also lead to osteonecrosis of the jaw (10). Previous studies have linked mandibular osteonecrosis to high-dose bisphosphonate (BP) or intravenous denosumab therapy, with the highest incidence (ranging from 1% to 15%) observed in cancer patients frequently exposed to elevated doses of these medications (10). However, this patient developed mandibular osteonecrosis after receiving only 5 mg of bisphosphonate over several years. However, further research is needed to explore the residual effects and side effects of bisphosphonates *in vivo*.

Surgical intervention becomes necessary when patients exhibit pathological fractures, severe bone deformities, arthritis, nerve compression, or other complications. Surgical procedures may include osteotomy and correction, complete lesion removal, bone grafting, internal fixation, and joint replacement to enhance the mechanical stability of the affected bone, alleviate symptoms, or restore functionality. The patient, who had endured femoral neck pain and limited mobility for an extended period, ultimately required total hip replacement. Nevertheless, patients with multiple FDs often exhibit a high rate of bone turnover, significant bone destruction, and osteoporosis, which significantly increases the risk of postoperative prosthesis loosening, thereby affecting long-term surgical outcomes. In a retrospective study by Sierra et al. (11) involving 27,543 patients who underwent total hip arthroplasty (THA), only 11 patients required replacement of a total of 12 hips due to FD. Of these, seven hips had cemented stems, while the remaining five had noncemented stems. Subsequently, seven hips required revision due to aseptic loosening during an average follow-up period of 12.5 years. In addition, three patients who had undergone noncemented stem replacement required earlier revision due to loosening (11). Notably, improvements have been made in prosthetics and surgical techniques, leading to lower complication and revision rates in patients implanted with noncement stems and conventional allograft materials. The reason behind this improvement is likely the advancements in prosthetics and surgical techniques (12). In this particular case, the patient received intravenous zoledronic acid before and after surgery to prevent aseptic loosening of the prosthesis. Bisphosphonates were used because they can reduce the bone conversion rate and increase bone mineral density in patients with fibrous dysplasia, thus slowing osteolysis around the prosthesis and reducing the risk of loosening after joint replacement. Postoperative monitoring revealed a gradual decrease in x-ray abnormalities, serum alkaline phosphatase levels, and osteocalcin levels. After 20 months, the x-ray film showed no signs of osteolysis around the prosthesis (Figure 2).

Stanton et al. (13) discovered that new bone that formed after fibrous dysplasia fracture and corrective osteotomy was dysplastic, meaning that recurrent fractures and malformations could occur. The femoral and tibial cortexes are typically severely damaged in most cases, discouraging the use of typical plates and screw devices unless screws can be placed outside the fibrous dysplasia lesion to obtain normal cortical bone. Lppolito et al. (14) conducted a multicenter study and found that intramedullary nail fixation was effective for treating femoral fibrous dysplasia with fracture. However, in patients with MAS, fractures and deformities are present, and intramedullary nail fixation is discouraged due to severe damage to the femoral cortex. In this case, plate screws were used to correct the deformity and fix the femoral shaft fracture.

In conclusion, this study acknowledges the challenges in diagnosing and treating MAS and emphasizes the importance of further research to determine optimal drug dosages and durations, as well as the selection of appropriate surgical methods to address bone quality and deformities. Prosthesis loosening caused by impaired bone growth after surgery is also highlighted as a challenge in patient rehabilitation.

## Data Availability

The original contributions presented in the study are included in the article/Supplementary Material, further inquiries can be directed to the corresponding author.
