# Supplementary figures and images for: A young woman with atypical McCune–Albright syndrome and the difficult road to recovery: a case report
Source: Front Surg. 2024 Feb 2;11:1326977. doi: 10.3389/fsurg.2024.1326977 (PMC10869435; doi:10.3389/fsurg.2024.1326977)

**skin pigmentation**

**
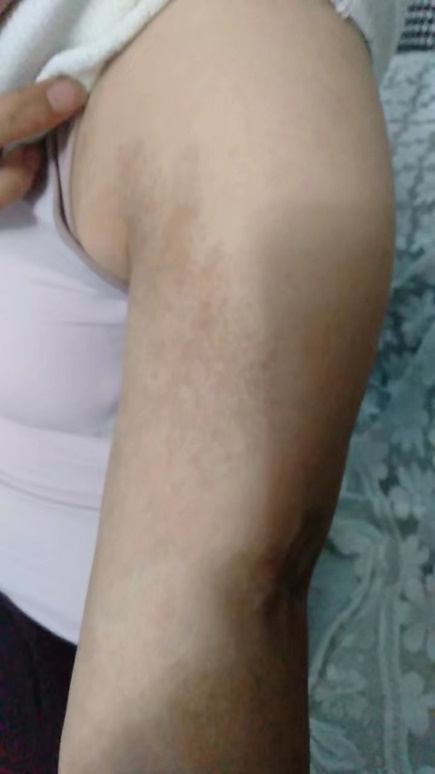
**

**PTH**


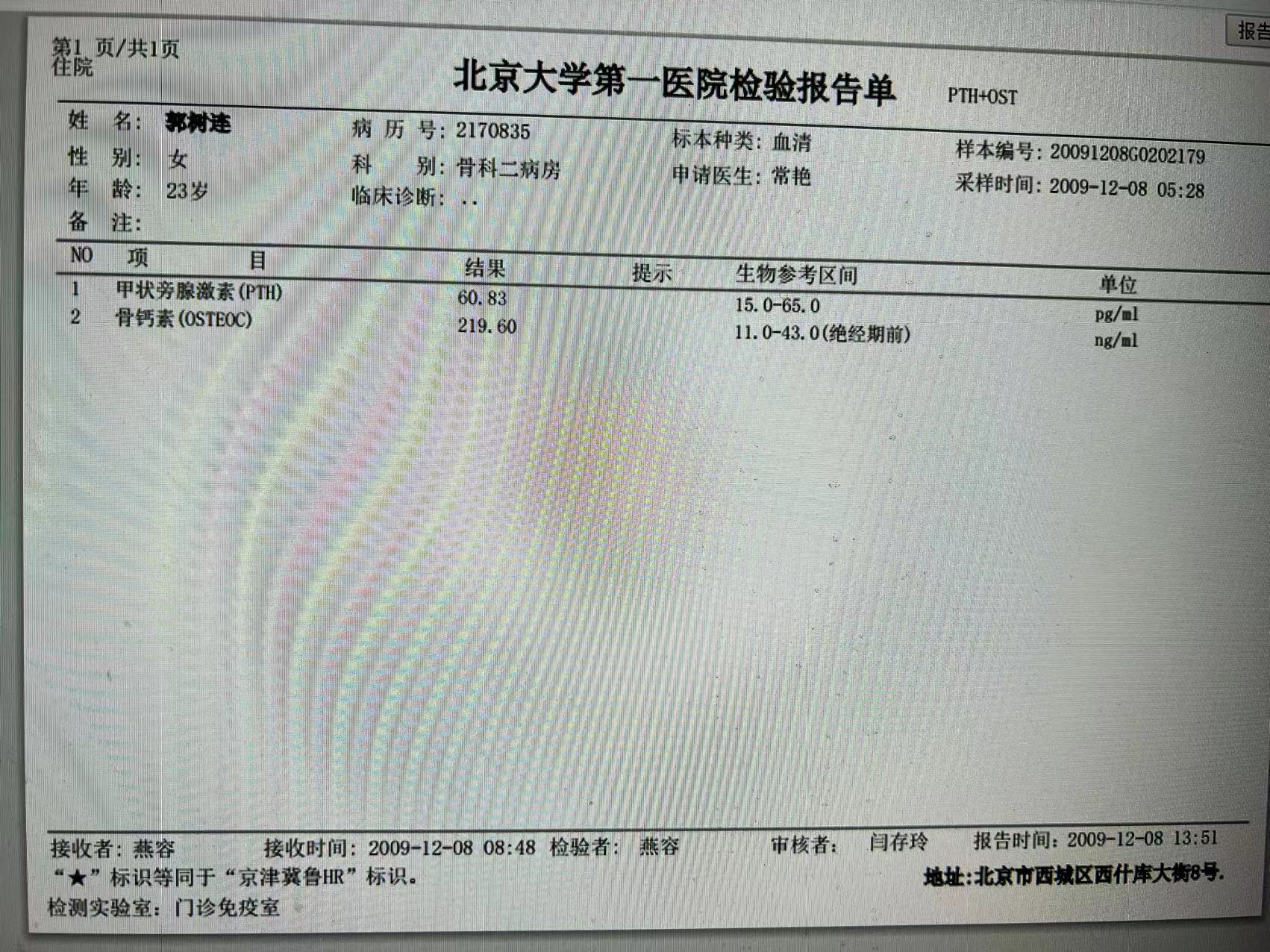


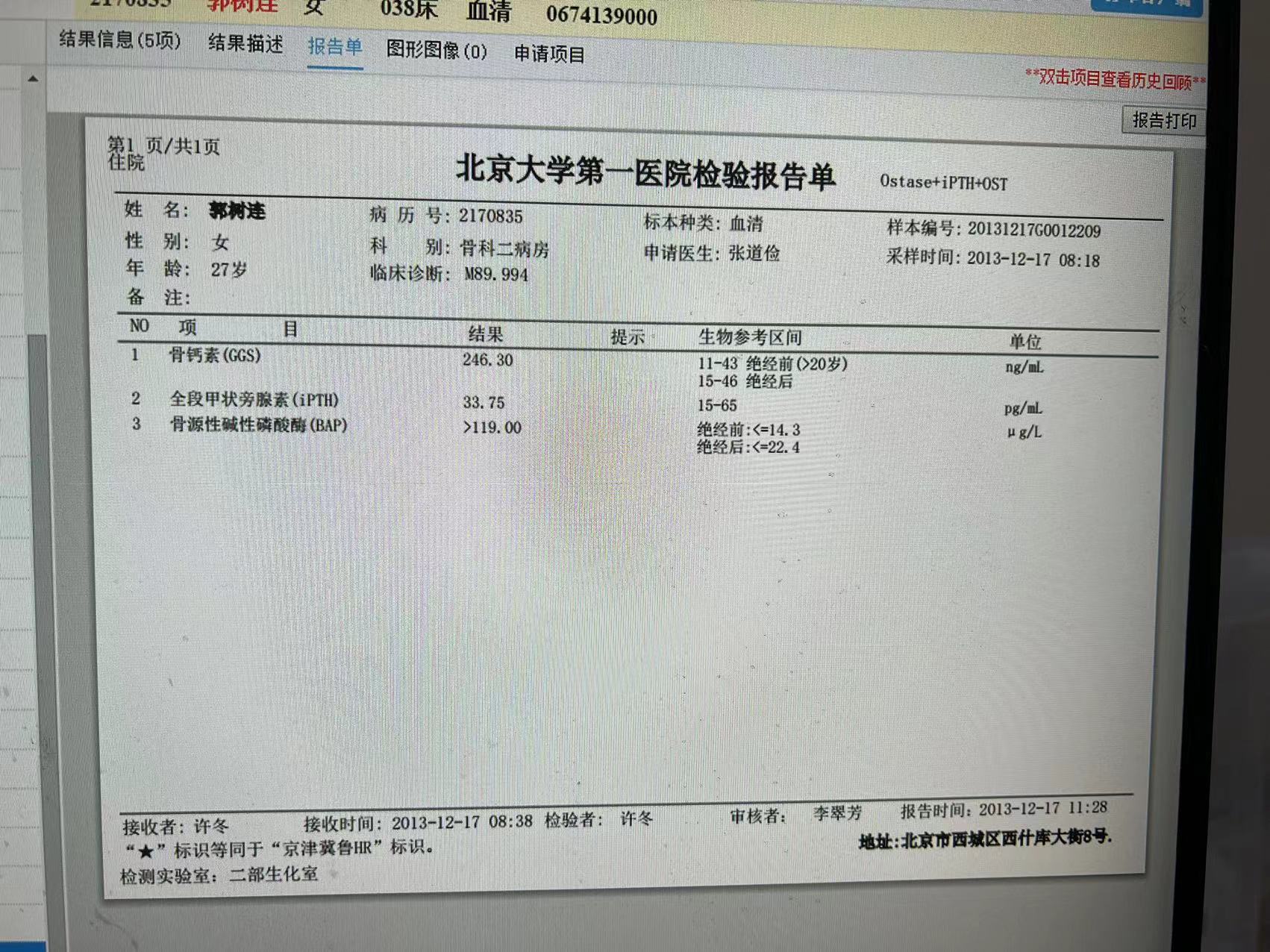


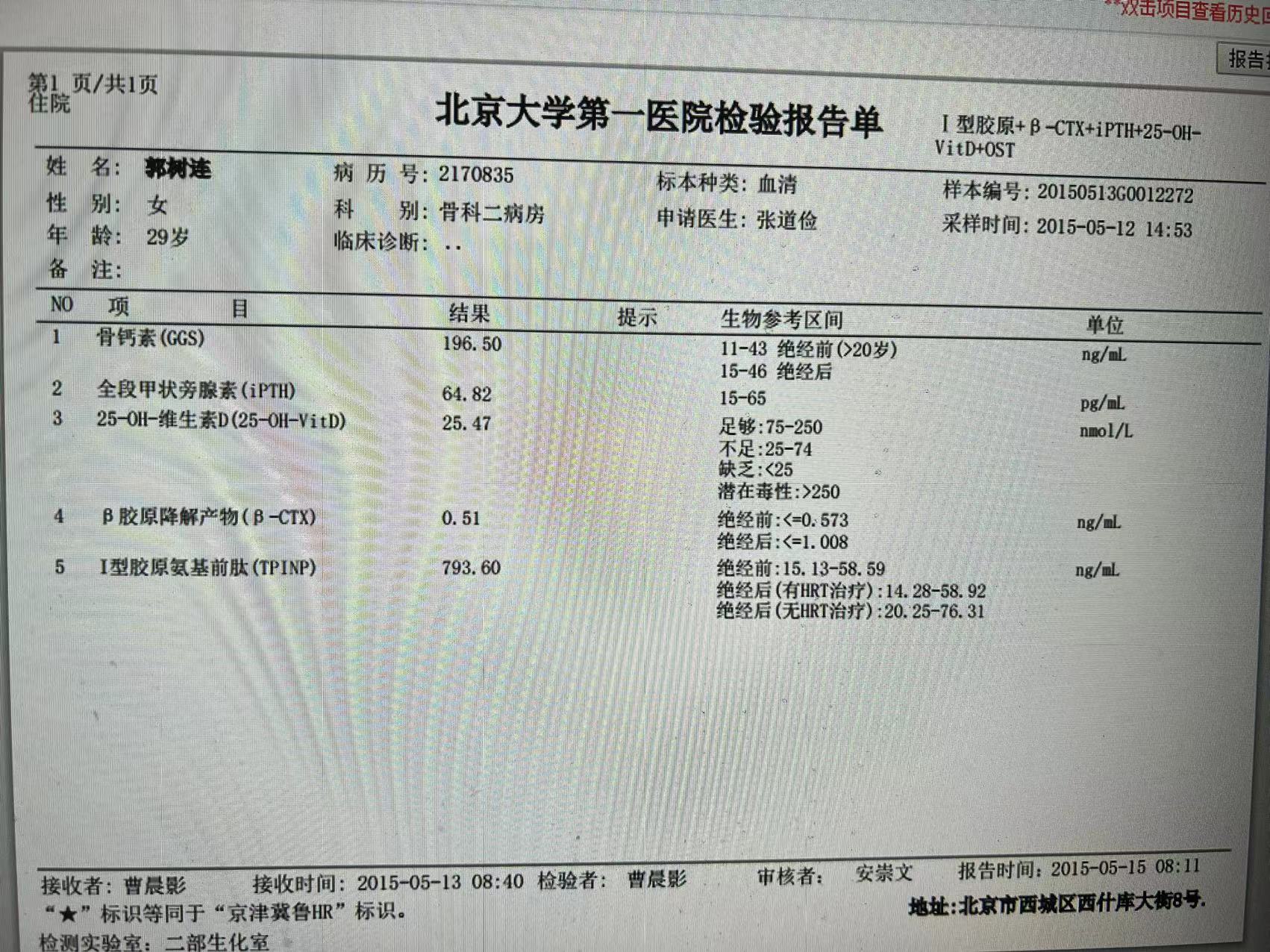


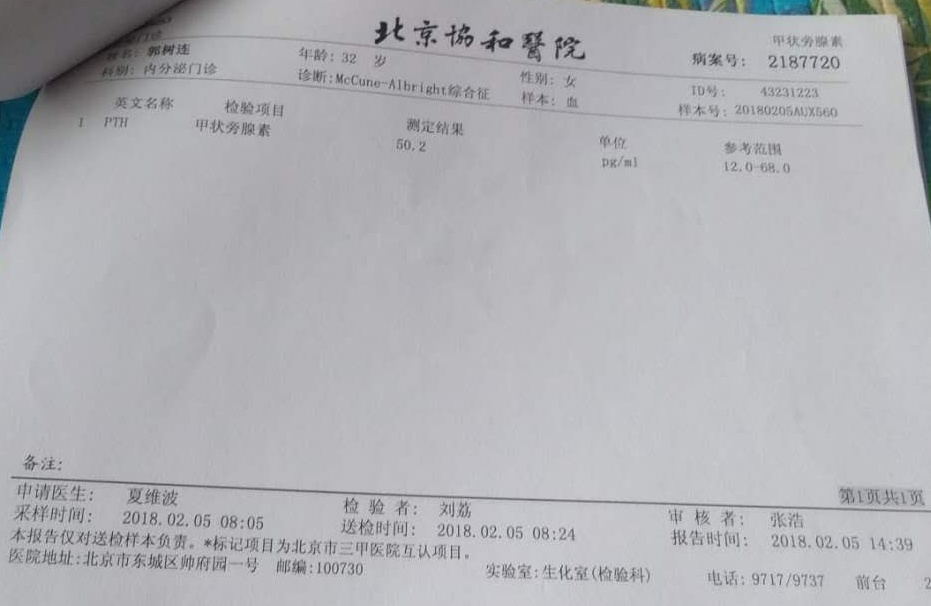


**ACTH**


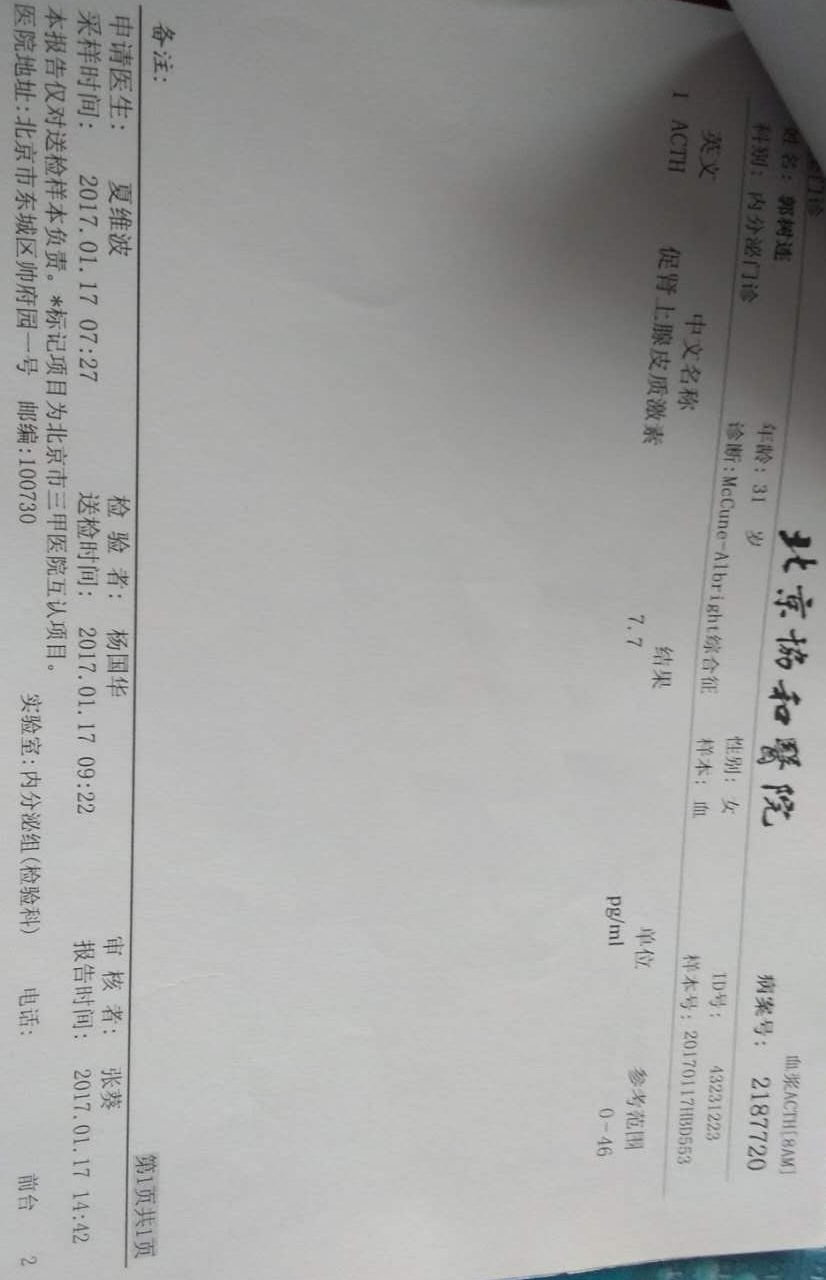


**cortisol**
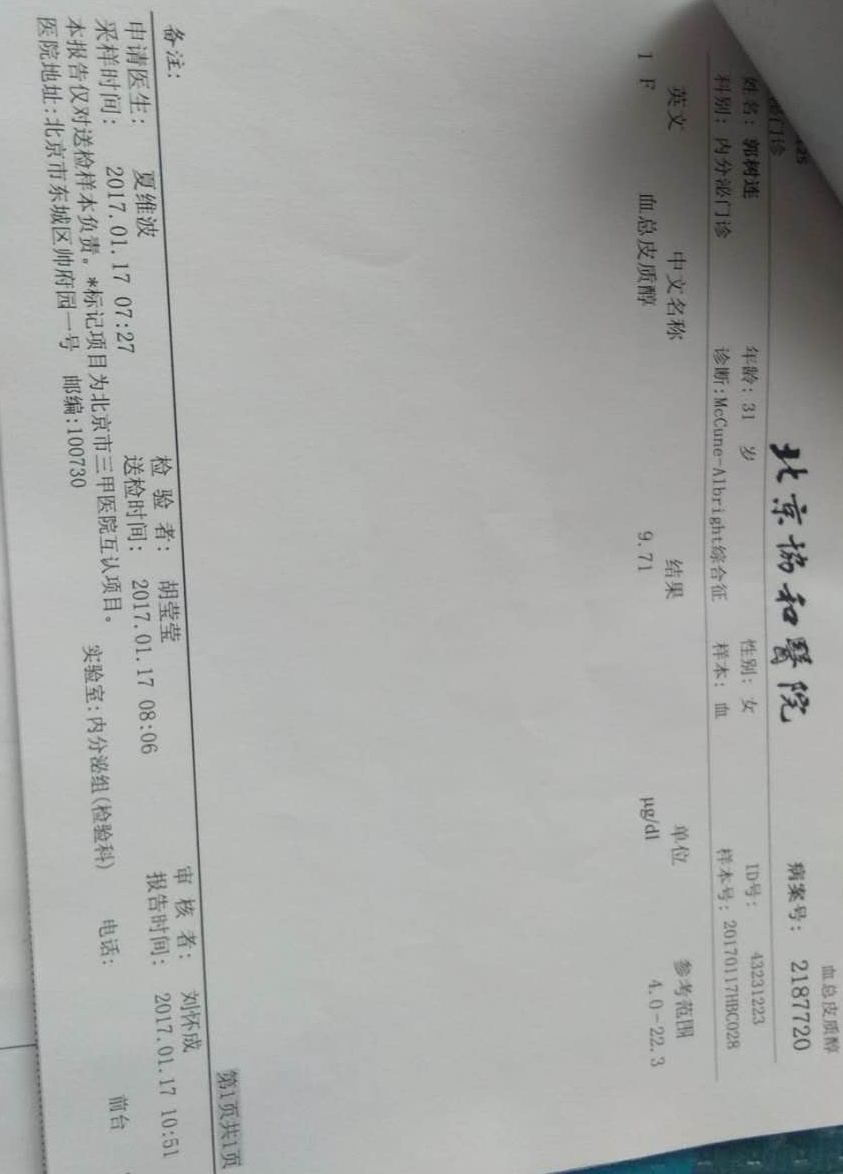


**thyroid function**


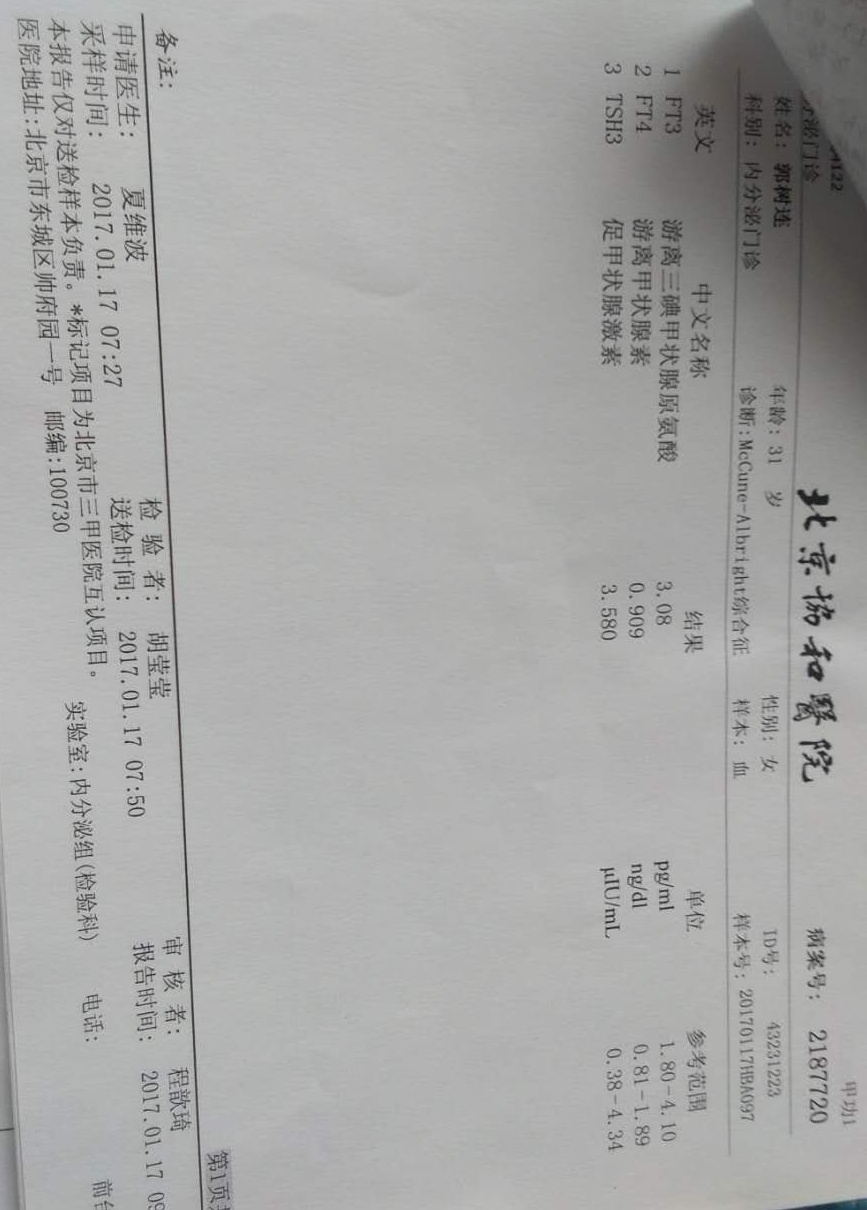


**IGF-1**


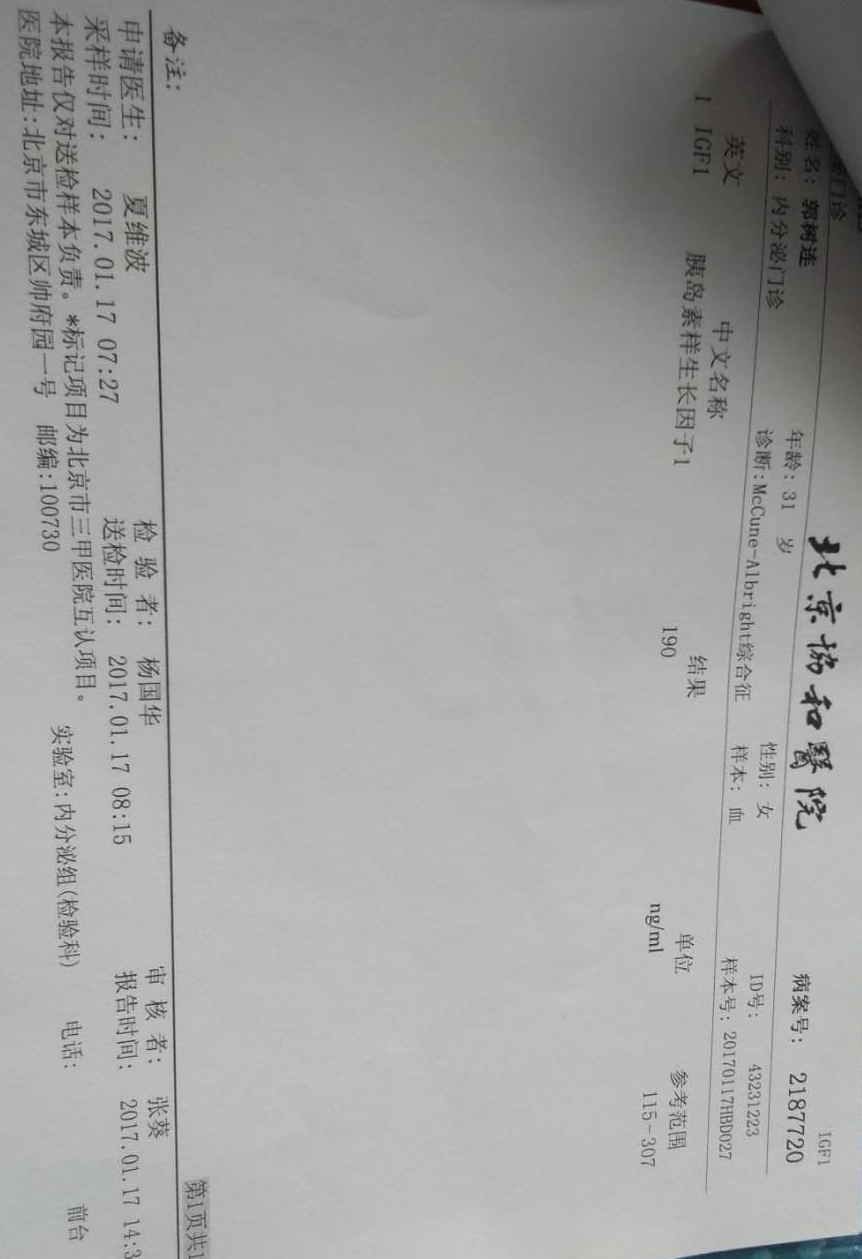

Supplement: Supplementary file 1 [file Datasheet1.docx]
